# Prevalence and risk factors for tobacco, khat, and alcohol consumption among high school students in Ethiopia

**DOI:** 10.1186/s12889-023-15088-x

**Published:** 2023-02-02

**Authors:** Selamawit Hirpa, Andrew W Fogarty, Adamu Addissie, Linda Bauld, Thomas Frese, Susanne Unverzagt, Eva J. Kantelhardt, Sefonias Getachew, Wakgari Deressa

**Affiliations:** 1 Department of Preventive Medicine, School of Public Health, Addis Ababa University, Addis Ababa, Ethiopia; 2 Division of Epidemiology and Public Health, University of Nottingham, Nottingham, UK; 3 Usher institute and SPECTRUM Consortium, University of Edinburgh, Edinburgh, UK; 4 General Practice and Family Medicine, Martin-Luther-University Halle-Wittenberg, Halle, Germany; 5 Institute for Medical Epidemiology, Biostatics and Informatics, Martin-Luther-University, Halle-Wittenberg, Germany; 6 Global Health Working Group, Martin Luther University Halle-Wittenberg, Halle (Saale), Germany

**Keywords:** Tobacco, Khat, Alcohol, High school, Students, Ethiopia

## Abstract

**Background:**

Tobacco, khat, alcohol, and marijuana are the main risk factors for non-communicable diseases. There are limited studies on substance use in Ethiopia, especially among secondary school students. This study aims to determine the epidemiology of substance use among secondary school students in Ethiopia.

**Methods:**

This cross-sectional study was conducted in March 2020 in four large regions of Ethiopia and the capital Addis Ababa. We collected data from 3,355 grade 9 and grade 10 students in 36 randomly selected high schools. Data were collected on tobacco, khat, alcohol and other substances. Mixed effect logistic regression models were fitted to determine the predictors of cigarette smoking.

**Results:**

157 (4.7%) of the participants ever smoked cigarettes and 81 (2.4%) were current smokers. 106 (3.2%) ever used smokeless tobacco, 1,342 (41.8%) had ever drunk alcohol, 290 (8.7%) ever used khat, 137 (4.8%) chewed khat regularly and 76 (2.3%) ever used marijuana. There was a significant regional variation in substance use patterns; cigarette and khat use was the highest in southern regions, whereas alcohol use was highest in the northern areas. Availability of cigarette and khat shops within a 100-meter radius of the school compound was reported by 1,229 (37.5%) and 816 (25%) students, respectively. Three hundred fifty-four (10.9%) students had ever seen someone smoking a cigarette in the school compound. Ever use of smokeless tobacco (Adjusted Odds Ratio (AOR) = 9.4, 95%CI: 4.9–17.9), ever use of shisha (AOR = 8, 95% CI: 3.9–16.3), ever use of khat (AOR = 4.1, 95%CI: 2.5–6.8), ever use of alcohol (AOR = 2.3, 95%CI: 1.4–3.7), having a friend who smoked a cigarette (AOR = 2, 95%CI: 1.2–3.5), and ever seen someone smoking a cigarette in the school compound (AOR = 1.9, 95%CI: 1.1–3.4) were associated with ever use of cigarettes.

**Conclusion:**

Substance use prevalence in Ethiopia has regional variations and prevention strategies should be tailored to the needs of the regions. Although this study reported a lower prevalence of cigarette smoking, students could access cigarettes and khat in nearby school areas. The existing tobacco control laws that prohibit selling tobacco products to children and adolescents under 21 years of age and ban establishing tobacco shops close to school compounds should be enforced.

**Supplementary Information:**

The online version contains supplementary material available at 10.1186/s12889-023-15088-x.

## Background

Tobacco, khat, alcohol, and marijuana are among the list of substances used by many globally and cause public health challenges due to their negative effects on the physical, mental, and social well-being of individuals who use them [1]. The World Health Organization (WHO) included tobacco and alcohol among the risk factors for four main non-communicable diseases (NCDs): chronic respiratory diseases, diabetes, cardiovascular diseases and most cancers [2]. Tobacco products are substances that are completely or partly made of tobacco leaf that can be administered through a variety of routes including smoking, chewing, sucking and nasal application as a snuff [3]. Tobacco smoke contains more than 7,000 chemicals and hundreds of these chemicals are proven to be hazardous to health [4].

Globally, an estimated 24 million children between the ages of 13 and 15 years use tobacco [5]. In Ethiopia, a previous study in Addis Ababa and the surrounding towns estimated a 3% prevalence of cigarette smoking among school-going adolescents [6]. These figures are low in comparison to studies conducted in other African countries [7], [8]. However, the existing large number of young population and the tobacco industry’s aspiration to create new markets in the country [9] laid the ground for a rapid future increase in consumption unless proper preventive measures are in place.

Khat is a plant containing a psycho-stimulant substance, and khat chewing is a pressing public health problem in East Africa. It is mostly chewed but it can also be infused as a tea or can be dried and smoked like a cigarette [10]. It is commonly grown in the region (Ethiopia, Kenya) and the Arabian Peninsula [11]. Studies have shown that khat consumption is associated with cardiovascular, gastrointestinal, mental, and oral hygiene problems [12], [13], and a cause for household economic instabilities [14]. In Ethiopia, khat is commonly used for social recreation, religious rituals and as a stimulant [15], [16], particularly by students aiming to improve academic performance. A study in eastern Ethiopia indicated that the mean age to start khat chewing was 15.1 years [10] and multiple substance use (khat, cigarette, and alcohol) was high [10].

Apart from the negative effects of khat on health, people who use it can also progress to cigarette smoking and alcohol use. The excessive use of alcohol is a major global contributor to morbidity, mortality, and injury. Among the health impacts of alcohol dependence, liver cirrhosis, cancers, and injuries are commonly reported [16]. Alcohol consumption is strongly associated with behavioral and mental disorders of youth. A meta-analysis that included studies conducted in Ethiopia between the years 2000 to 2019 reported that regular alcohol consumption among high school students was 23% [17]. Marijuana use among adolescents is linked with mood disorders, poor academic performance, psychotic and addiction problems [18]. Limited studies exist about the prevalence of marijuana use in Ethiopia. A study conducted in the year of 2011 in the southern part of Ethiopia showed that 0.9% of university students used marijuana in the past 12 months [19].

Ethiopia is the second- most populous country in Africa with more than 115 million people of which 60% are under the age of 25 years [20]. Ethiopia is the largest producer of khat in the world, and it is one of the largest export commodities with annual earnings of more than one hundred million dollars [21], [22]. In Ethiopia, most previous studies on substance use were limited to mostly khat use among university students living in not more than one region [10], [14], 15], [23]. The current study included four big regions and the capital Addis Ababa. This makes it the first study to cover a wide geographic area and a range of substances among secondary school students. Accurate estimates of substance use are critical to designing prevention and control strategies. Therefore, this study aimed to estimate the prevalence and risk factors of tobacco, khat, and alcohol use among school-going adolescents in Ethiopia.

## Methods

This study draws on data from a survey described in a previously published paper on shisha smoking among school-going adolescents in Ethiopia [24].

### Study setting and design

Ethiopia has 11 regions and two chartered cities. The official secondary school age is between 15 and 18 years. The national Gross Enrollment Ratio (GER) for grades 9–12 was 32% in the year 2018/2019 with Addis Ababa (the capital city) having the highest with 87.6% [25]. This study was conducted in the main cities (i.e., Adama, Bahir Dar, Hawassa, Mekelle, and Addis Ababa) and peri-urban (within a 50 km radius of the main cities) areas of the four large regions.

### Study population and sample size calculation

School-aged adolescents 13–22 years who were in grades 9 and 10 participated in the study. Open Epi Version 7.02 statistical software [26] was used to calculate the minimum sample size. The sample size was calculated to estimate the prevalence of cigarette, shisha, khat use, and alcohol consumption [27] with a 95% precision, 80% power and an Intra-cluster Correlation Coefficient (ICC) of 0.1. 20% was added for possible non-response for the pupils and 10% for the schools. The minimum sample size was 4,714 clustered into 50 schools. However, due to the COVID-19 pandemic and subsequent school closures, in March 2020, we were able to collect data only from 36 out of the 50 schools. The total number of schools included from urban and peri-urban study areas was 6 from Adama, 5 from Hawassa, 9 from Bahir Dar, 10 from Mekelle, and 6 from Addis Ababa. The respective number of study participants were 585 from Adama, 557 from Hawassa, 874 from Bahir Dar, 881 from Mekelle and 458 from Addis Ababa.

### Sampling procedures

To produce a representative sample of study participants, a two-stage cluster sampling approach was employed. The total number of schools in the sampling frame was four hundred fifty-five (455). For the selected urban and peri-urban areas, of each region, two sampling frames were prepared separately, which included the list of government and private schools, and the study schools were randomly selected from these frames. The secondary sampling units were class sections for grades 9 and 10 in each school. The mean number of sections in preschools was 9.5. All students in the selected class section were invited to participate in the study and the sampling probability was 1.5%.

### Survey instruments and data collection

Interview questionnaire was used to assess substance use and its risk factors among students. School directors were interviewed about the availability of cigarette and khat shops surrounding the school compound. The tools were developed by adapting the Global Youth Tobacco Survey (GYTS) [28] and the Youth Risk Behavior Survey (YRBS) instruments and by reviewing relevant literature. The tools were translated from English to 3 local languages (Amharic, Afan Oromo, and Tigrigna) and were pre-tested among 250 students. Data collection was conducted from March 9 to 17, 2020 after giving a 2-days training for data collectors.

Consent was obtained from each schoolmaster to collect data from students in the regular class session in the absence of their teachers or school directors. Students in the selected classes were asked to complete the paper-based questionnaire individually and to consult the data collectors for any ambiguity on the questions. Due to the COVID-19 pandemic, we could not collect data from all the selected 50 schools.

### Data analysis

After data were checked for completeness and consistency, Epidata Version 3.1 software [29] was used to enter the data. Data were analyzed using Stata/SE 14.0 software [30]. The survey weight was calculated considering the total and the selected schools in the study areas, the total number of grade 9 and 10 students in the study areas and the number of students who participated in the study. To determine our survey weight, we used STATA command, [generate weight_ cluster = (FPC1*FPC2) ^-1]. After that, we set our survey data using STATA command “svyset PSU [pweight = weight_cluster], fpc (FPC1)||SSU, fpc(FPC2) singleunit(centered). The primary sampling unit (PSU) for our analysis was schools and the secondary sampling unit (SSU) was class sections and all pupils in the selected sections were invited to participate in the study. The primary outcome variable of this study was ever smoking cigarettes. Independent variables in the level- 1 model were socio-economic variables and ever use of different substances. Independent variables in model-2 included all the 11 variables in model-1 and an additional 4 school-level variables.

A two-level mixed-effects logistic regression model was fitted to adjust for confounding variables and account for school level clustering. The model fitting was a three-step process where the null model (model-0) measured the random effect and ICC in the odds of ever smoking cigarettes. The ICC represented the proportion of the within school variation on ever smoking cigarette in the total variation [31]. The first model (model-1) was fit to assess the effect of student-level predictors on the odds of ever smoking cigarettes. The final model (model-2) was fit to assess the effect of student-level characteristics and cluster level (school level) factors on ever smoking cigarettes. The *xtmelogit* STATA command was used to run the models. Crude odds ratios (OR) and adjusted OR (AOR) with their corresponding 95% confidence intervals (CIs) were calculated using univariate logistic and multivariable mixed-effects logistic regression models, respectively. The level of statistical significance was set at α = 0.05.

## Results

A total of 3,457 students were invited to participate in the study from 36 schools (82% of the targeted 50 schools) and 3,355 (97%) participated in this study. Data were analyzed for 3,347 students after discarding eight questionnaires that were incomplete for most of the variables. In this study, 1,804 (54%) were females. The age of 3,001 (89.7%) students was between 15 and 18 years and the mean age (± SD) was 16.5 ± 1.4 years. Of study participants who ever used cigarettes, 89 (57.5%) started smoking in the age between 12 and 16 years. A total of 2,618 (78%) students were from government schools. Additional information on students’ socio-demographic characteristics is described elsewhere^24^.

### Prevalence of substance use among high school students in Ethiopia

A total of 157 (4.7%) of students ever used cigarettes and 81 (2.4%) were regular cigarette smokers. The prevalence of ever use and regular use of khat was 290 (8.7%) and 137 (47.9%), respectively. We found variations in the prevalence of substance use between regions (Table 1). The highest proportion of cigarette smoking, khat chewing and ever use of smokeless tobacco was reported from Hawassa, southern Ethiopia. 1342 (40.1%) of students ever drank alcohol and the highest prevalence (69.4%) was from Bahir Dar, northwestern Ethiopia. We found that 76 (2.3%) of students used marijuana and the highest proportion was from Addis Ababa (5.8%).

**Table 1:** Weighted prevalence of substance use among high school students in Ethiopia (March 2020)

Variables	Categories	Frequency (%)	[95% CI*]
Ever use of cigarette (n = 3,347)	Overall	157 (4.7)	[3.3–6.7]
	Addis Ababa	34 (7.4)	[5.8–9.5]
	Adama	24 (4.1)	[2.2–7.6]
	Bahir Dar	22 (2.5)	[1.4–4.5]
	Hawassa	64 (11.4)	[7.7–16.5]
	Mekelle	13 (1.5)	[0.8–2.8]
Current use of cigarette (n = 3,347)	Overall	81(2.4)	[1.7–3.5]
	Addis Ababa	16 (3.5)	[2.2–5.6]
	Adama	13 (2.2)	[1.0–5.0]
	Bahir Dar	11(1.3)	[0.5-3.0]
	Hawassa	34 (6.1)	[4.2–8.7]
	Mekelle	7 (0.9)	[0.4–1.7]
Ever drink alcohol products (n = 3,347)	Overall	1342 (40.1)	[32.3–48.5]
	Addis Ababa	162 (35.4)	[30.4–40.7]
	Adama	145 (24.7)	[17.8–33.2]
	Bahir Dar	607 (69.4)	[57.5–79.2]
	Hawassa	125 (22.3)	[15.1–31.7]
	Mekelle	303 (34.9)	[24.9–46.6]
Regularly drink alcohol (n = 1,347)	Overall	559 (41.5)	[34.6–48.7]
	Addis Ababa	51 (31.5.0)	[18.7–47.9]
	Adama	37 (25.2)	[16.7–36.1]
	Bahir Dar	248 (40.7)	[29.8–52.8]
	Hawassa	81 (64.8)	[50.2–77.1]
	Mekelle	142 (46.6)	[32.1–61.6]
Ever use of khat (n = 3,347)	Overall	290 (8.7)	[6.6–11.3]
	Addis Ababa	60 (13.1)	[10.5–16.2]
	Adama	68 (11.6)	[9.8–13.7]
	Bahir Dar	79 (9.0)	[6.1–13.1]
	Hawassa	75 (13.4)	[7.9–21.8]
	Mekelle	8 (0.9)	[0.3–2.4]
Regular use of khat (n = 286)	Overall	137 (47.9)	[42.1–53.7]
	Addis Ababa	21 (35.6)	[13.2–66.7]
	Adama	32 (47.1)	[37.2–57.1]
	Bahir Dar	30 (38.5)	[29.1–48.8]
	Hawassa	51(68.0)	[50.3–81.7]
	Mekelle	3 (50.0)	[8.4–91.6]
Ever use of smokeless tobacco (n = 3,346)	Overall	106 (3.2)	[2.0–5.0]
	Addis Ababa	17 (3.7)	[2.1–6.5]
	Adama	18 (3.1)	[1.9–4.9]
	Bahir Dar	10 (1.1)	[0.4–3.6]
	Hawassa	37 (6.6)	[4.3–10.1]
	Mekelle	24 (2.8)	[1.3–5.9]
Ever use of marijuana (n = 3,244)	Overall	76 (2.3)	[1.6–3.1]
	Addis Ababa	26 (5.8)	[4.5–7.1]
	Adama	12 (2.0)	[1.5–2.8]
	Bahir Dar	18 (2.1)	[1.4-3.0]
	Hawassa	18 (3.3)	[1.7–6.1]
	Mekelle	2 (0.24)	[0.0-1.6]

*Confidence Interval

### Socio demographic characteristics of substance users

From students who ever used cigarettes 114 (73.5%) were males (Table  2). More than 68.2% of students who ever used cigarettes were in the age group of 15–17 years. Three hundred fifty-four (10.9%) students had ever seen someone smoking cigarettes in the school compound. Almost 74.7% of regular alcohol drinkers consumed homemade beer (*Tella*).

**Table 2:** Weighted proportion of substance use by socio-demographic characteristics of high school students in Ethiopia (March 2020**)**

Variables	Categories	Frequency (%)	[95% CI]
Sex proportion of participants who ever used cigarettes (n = 155)	Male	114 (73.5)	[65.9–80.3]
	Female	41 (26.5)	[19.7–34.1]
Proportion by age intervals of participants who ever used cigarettes (n = 157)	13–14	3 (1.9)	[0.5–5.1]
	15–17	107 (68.2)	[60.6–75.1]
	18–19	41 (26.1)	[19.7–33.4]
	20 and above	6 (3.8)	[1.6–7.8]
Age at initiation of cigarette smoking (n = 123)	≤ 11 years	17 (13.8)	[8.5–20.8]
	12–14 years	41 (33.4)	[25.4–42.0]
	15–16 years	48 (39.0)	[30.7–47.9]
	17 and above	17 (13.8)	[12.7–15.0]
Ever seen anyone smoking in the school (n = 3,261)	No	2,254 (69.1)	[63.1–73.0]
	Yes	354 (10.9)	[6.9–16.4]
	I am not sure	653 (20)	[18.7–21.4]
Regular alcohol drinkers consume (n = 559) (More than one response was possible)	Beer	196 (36)	[31.1–39.2]
	Wine	99 (18.1)	[14.7–21.0]
	Local drink (Areke)	106 (19.4)	[15.9–22.4]
	Local honey wine (*Tej*)	95 (17.4)	[14.1–20.3]
	Homemade beer (Tella)	408 (74.7)	[69.2–76.6]

### Cigarette smoking status of friends and parents

Three hundred sixty (11%) study participants reported that they had friends who smoked cigarettes. One hundred thirty-five (38.4%) of them acknowledged that their friends smoked every day (Table 3). More than 90% of the students’ fathers and mothers did not smoke cigarettes.

**Table 3:** Weighted prevalence of cigarette smoking practice of friends and parents of the study participants (March 2020)

Variables	Categories	Frequency (%)	[95% CI]
Any of your friends’ smoke cigarettes (n = 3,277)	No	2,389 (72.9)	[71.4–74.4]
	Yes	360 (11.0)	[9.9–12.1]
	I don’t know/I am not sure	528 (16.1)	[14.9–17.4]
How often do your friends smoke cigarettes (n = 352)	Every day	135 (38.4)	[33.4–43.5]
	Weekly	25 (7.1)	[4.8–10.2]
	Sometimes	94 (26.7)	[22.3–31.5]
	I don’t know/I am not sure	98 (27.8)	[23.4–32.7]
Father smokes cigarettes (n = 3,277)	No	2,969 (90.6)	[89.6–91.6]
	Yes	60 (1.8)	[1.4–2.3]
	I don’t know	248 (7.6)	[6.7–8.5]
Mother smokes cigarettes (n = 3,277)	No	3,125 (95.4)	[94.6–96.0]
	Yes	5 (0.2)	[0.1–0.3]
	I don’t know	147 (4.5)	[3.8–5.2]

### Access to cigarettes and khat retail outlets

Of the students, 851 (26.0%) had ever gone to the shop to buy cigarettes and 206 (24.6%) of them reported that it was easy to buy cigarettes (Table 4). A total of 1,229 (37.5%) students acknowledged that there were shops for cigarettes within 100 m radius of the school. Of the interviewed 36 school directors, 22 (61.1%) responded that there were shops for cigarettes within a 100-meter radius from the schools. 816 (25.0%) of students mentioned that there were khat shops within a 100-meter radius of the school compound.

**Table 4:** Access to cigarettes and khat retail outlets among high school students in Ethiopia (March 2020)

Variables	Categories	Frequency (%)	[95% CI]
Ever gone to the shop to buy cigarettes? (n = 3,274)	No	2,423 (74.0)	[72.5–75.5]
	Yes	851 (26.0)	[24.5–27.5]
Buying cigarettes from a shop? (n = 837)	Very difficult	327 (39.1)	[35.8–42.4]
	Difficult	145 (17.3)	[14.8–20.0]
	Easy	206 (24.6)	[21.8–27.6]
	Very easy	159 (19.0)	[16.5–21.8]
Where do you get cigarettes from? (n = 851) (More than one answer was possible)	Supermarket	133 (12.9)	[10.9–15.0]
	Petty trader	649 (62.8)	[59.9–65.7]
	Street market	171 (16.6)	[14.4–18.9]
	Friends/Family/ Teachers	29 (2.8)	[1.9-4.0]
	Other	51 (4.9)	[3.7–6.4]
Are there any cigarette shops within 100 m radius from your school? (n = 3,274)	No	909 (27.8)	[26.3–29.3]
	Yes	1,229 (37.5)	[35.8–39.2]
	I don’t know	1,136 (34.7)	[33.1–36.3]
Do students buy cigarettes from shops that are found in 100 m radius from the school compound? (n = 1,205)	No	148 (12.3)	[10.5–14.2]
	Yes	297 (24.7)	[22.3–27.1]
	I don’t know	760 (63.0)	[60.3–65.8]
*Are there cigarette shops within 100 m radius from the school compound? (n = 36)	No	14 (38.9)	[24.1–55.4]
	Yes	22 (61.1)	[44.6–75.9]
Are there khat shop within 100 m radius from the school compound? (n = 3,260)	No	1,186 (36.4)	[34.7–38.0]
	Yes	816 (25.0)	[23.6–26.5]
	I don’t know	1,258 (38.6)	[36.9–40.3]
Do students chew khat in shops that are found in 100 m radius from the school compound? (n = 795)	No	89 (11.1)	[9.1–13.5]
	Yes	188 (23.7)	[20.7–26.7]
	I don’t know	518 (65.2)	[61.8–68.4]

*Response by school directors

### Individual and school-level variables associated with ever smoking cigarettes

Significantly associated variables with ever use of cigarettes, in model-1 and model-2 were similar (Table 5). Furthermore, one variable from the school level variables was significantly associated with ever use of cigarettes. Ever use of smokeless tobacco (AOR = 9.4, 95%CI: 4.9–17.9), ever smoked shisha (AOR = 8.0, 95% CI: 3.9–16.3), ever used khat (AOR = 4.1, 95%CI: 2.5–6.8), ever drank alcohol (AOR = 2.3, 95%CI:1.4–3.7), friends smoked cigarette (AOR = 2.0, 95% CI: 1.2–3.5), and ever seen anyone smoking cigarette in the school (AOR = 1.9, 95% CI:1.1–3.4) were strongly associated with ever use of cigarettes.

**Table 5:** Multi-level logistic regression model for ever use of cigarettes among high school students in Ethiopia (March 2020)

Variables	Categories	Ever smoked cigarette (%) n = 157	Model-1 OR (95% CI)	Model-2 AOR (95% CI)
Age	13–17	108	-	-
	18 and above	45	0.9 [0.6–1.5]	0.9 [0.5–1.5]
Sex	Male	112	-	-
	Female	39	0.6 [0.4-1.0]	0.6 [0.4-1.0]
Parent’s residence	Rural	42	-	-
	City	111	1.4 [0.8–2.3]	1.3 [0.8–2.3]
Source of income from parents	No	29	-	-
	Yes	124	1.2 [0.6–2.3]	1.2 [0.6–2.3]
Ever use of khat	No	71	-	-
	Yes	82	**4.4 [2.7–7.1]**	**4.1 [2.5–6.8]**
Ever use smokeless tobacco	No	104	-	-
	Yes	49	**8.7 [4.6–16.4]**	**9.4 [4.9–17.9]**
Ever drink alcohol	No	43	-	-
	Yes	110	**2.3 [1.4–3.7]**	**2.3 [1.4–3.7]**
Ever used marijuana	No	118	-	-
	Yes	33	1.5 [0.7–3.5]	1.4 [0.6–3.2]
Ever smoked shisha	No	101	-	-
	Yes	51	**7.8 [3.9–15.7]**	**8.0 [3.9–16.3]**
Any of your friends smoke a cigarette	No	59	-	-
	Yes	76	**2.4 [1.4-4.0]**	**2.0 [1.2–3.5]**
	I don’t know	18	1.1 [0.6–1.9]	1.0 [0.5–1.9]
Father smokes cigarette	No	129	-	-
	Yes	6	2.2 [0.8–6.4]	2.2 [0.8–6.3]
	I do not know	18	1.3 [0.7–2.5]	1.2 [0.6–2.3]
School type	Government	120		-
	Private	33		1.2 [0.7-2]
Ever seen anyone smoking a cigarette in the school	No	81		-
	Yes	48		**1.9 [1.1–3.4]**
	I don’t know	23		1.1 [0.6-2]
Cigarette shops within 100 m radius	No	46		-
	Yes	107		1.2 [0.7-2]
Number of students in the school				1.1 [0.9–1.3]

(-) Reference group

Model-1 includes student-level characteristics as socio-economic variables (age, sex, parent’s residence, source of income) and ever use of different substances (khat, smokeless tobacco, alcohol, marijuana, shisha, any of your friends and/or father smoke a cigarette).

Model-2 includes student-level and school-level-characteristics (school type, ever seen anyone smoking cigarette in the school, cigarette shops, number of students in the school).

## Discussion

While cross-sectional surveys regularly provide data on substance use in adolescents in high income countries, there remain relatively few reports of this kind in low-income and middle-income countries including the countries in East Africa region. Ever and regular cigarette smoking prevalence is 4.7% and 2.4%, respectively. 40% of students had ever drunk alcoholic products and 8.7% had ever used khat. A small proportion (3.2%) of participants had ever used smokeless tobacco and 2.3% had ever used marijuana. Ever use of smokeless tobacco, shisha, khat, alcohol, and friends who smoked cigarettes were individual participant level factors that were significantly associated with ever use of cigarette. From the school-level variables, ever seeing anyone smoking in the school was significantly associated with ever use of cigarettes.

In Ethiopia therefore, substance use prevalence has a wide variation across regions due to accessibility, social norms, geographic, religious, and socio-economic factors [32]. We reported differences in the prevalence of cigarettes, alcohol, khat, smokeless tobacco and marijuana in different regions. Ever use of cigarette, smokeless tobacco and khat were highest in Hawassa, southern Ethiopia. This could be explained by wide khat cultivation in the region, also a common practice of concurrent use of khat chewing and cigarette smoking [33], [34]. Studies that explore the culture and perceived health risks in relation to smokeless tobacco are needed to understand the higher prevalence in Hawassa. Our study finding of ever use of khat in this region (13.4%) is comparable to the study conducted in the same region (14.6%) in the year 2015 [35].

The lowest rate of cigarette and khat use in Mekelle, northern Ethiopia, reported in this study could be described as the region being the first in banning khat cultivation and consumption. Moreover, cigarette smoking in public places was banned in 2015 [32]. In Ethiopia, there are national laws regarding preventive measures for the use of tobacco products and alcohol. This is encouraging; however, it takes more attention to enforce the law both in the capital and the regions.

Our study reported a lower prevalence of current use of cigarettes compared to studies conducted in Ghana, Uganda, and other parts of Ethiopia [7], [8], [10], [36]. Studies conducted in other parts of Ethiopia were specific to one city or town where the problem of cigarette smoking or other substances, such as khat chewing, was common which could explain the higher prevalence of cigarette use.

In this study, school-going adolescents who use khat were 4.1 times more likely to ever use cigarettes. Studies conducted in the adult population and adolescent age groups reported that khat chewing is a gateway to tobacco products [32], [34], [37]. This study also found that students who ever used smokeless tobacco, shisha and drank alcohol had higher odds of ever use of cigarettes. Once students are exposed to one substance, it is likely that they would experiment with new substances leading to concurrent use of substances.

Having a friend who smokes cigarettes was significantly associated with the ever use of cigarettes and this agrees with studies conducted in other parts of Ethiopia and other countries [6], [33], [38], [39]. This could be the results of peer influence or having a higher chance that adolescents would have friends with similar experiences. To minimize peer influence, awareness programs about the health effects of substance use should be designed for schools. Moreover, students who had ever seen anyone smoking cigarette in the school were significantly associated with ever use of cigarette. Under normal circumstances, it is not common to see someone smoking in school compounds. However, students smoke in hiding places like at the back of toilets in schools and those who ever smoked cigarettes had a higher chance of reporting smoking in these places and see someone smoking.

Based on the existing tobacco control law in Ethiopia (proclamation 1112/2019) [40], selling tobacco products to adolescents and youth aged younger than 21 years is prohibited. Likewise, smoking in school compounds and selling tobacco products within 100 m premise from the school compound is also not allowed. However, more than 60% of school directors who took part in this study and almost 40% of students reported that there were cigarette shops within 100 m radius of the school compounds. 11% of students saw someone smoking in school compounds at least once and 26% ever went to a shop to buy cigarettes. This suggests a gap in the implementation of the tobacco control law in Ethiopia. Tobacco control law enforcers should give priority and design a system to make sure school environments are free from tobacco products and other substances. Also, schools should play a major role in the implementation of a smoke-free environment in schools and their surroundings.

### Strengths and limitations

The study involved a range of substances and a wide geographic area. This enabled to generate a nationally representative data for multiple substances. However, the study is not without limitations, the cross-sectional study design provides a snapshot of substance use prevalence rather than exploring causal relationships. Second, social norms may mask the true prevalence of cigarette and other substance use. To minimize this, school directors and teachers were not present in the class at the time of data collection. Finally, even though the response rate was high, we collected data from eligible students who were attending classes at the time of the survey. This could have affected our estimates considering the possible association between substance use and poor school attendance. Since we have analyzed the data at the school and individual student levels, we have only accounted for school level correlation. This might undermine the power of the study in determining predictors of ever use of cigarettes. We haven’t conducted non-response adjustment as well, however, since the response rate was considerably high, we don’t expect this will have an impact on the study outcome.

## Conclusion

Ever use and current smoking of cigarettes among school-going adolescents in Ethiopia were generally low and varied across regions. Cigarette, khat, smokeless tobacco and marijuana use were most prevalent in Hawassa, southern Ethiopia, and Addis Ababa. Whereas almost 70% of students from Bahir Dar, northern Ethiopia ever used alcohol. Cigarette shops were available within 100 m radius from more than half of the studied schools. Control measures should be taken to ensure that school environments are free from cigarettes, khat retailers. Like tobacco, there needs to be a law to control the pervasive khat chewing practice of adolescents. In addition, tobacco and alcohol control law enforcement should be strengthened and substance use prevention and control programs should be available in schools.

## Electronic supplementary material

Below is the link to the electronic supplementary material.

Supplementary Material 1

## Data Availability

All data generated or analyzed during this study are included in this published article and its supplementary information files.

## List of abbreviations

AOR: Adjusted Odds Ratio
CI: Confidence Interval
GER: Gross Enrollment Ratio
GYTS: Global Youth Tobacco Survey
ICC: Intra-Cluster Correlation Coefficient
IRB: Institutional Review Board
OR: Odds Ratio
NCDs: Non-Communicable Diseases
PSS: Primary Sampling Unit
SD: Standard Deviation
SPH: School of Public Health
SSU: Secondary Sampling Unit
WHO: World Health Organization
YRBS: Youth Risk Behavior Survey
